# Bioactive Properties of a Novel Antibacterial Dye Obtained from Laccase-Mediated Oxidation of 8-Anilino-1-naphthalenesulfonic Acid

**DOI:** 10.3390/molecules27020487

**Published:** 2022-01-13

**Authors:** Jolanta Polak, Marcin Grąz, Kamila Wlizło, Katarzyna Szałapata, Justyna Kapral-Piotrowska, Roman Paduch, Anna Jarosz-Wilkołazka

**Affiliations:** 1 Department of Biochemistry and Biotechnology, Institute of Biological Sciences, Maria Curie-Skłodowska University, 20-031 Lublin, Poland; marcin.graz@mail.umcs.pl (M.G.); katarzyna.szalapata@mail.umcs.pl (K.S.); anna.jarosz-wilkolazka@mail.umcs.pl (A.J.-W.); 2 Department of Industrial and Environmental Microbiology, Institute of Biological Sciences, Maria Curie-Skłodowska University, 20-031 Lublin, Poland; kamila.wlizlo@mail.umcs.pl; 3 Department of Functional Anatomy and Cytobiology, Institute of Biological Sciences, Maria Curie-Skłodowska University, 20-031 Lublin, Poland; justyna.kapral-piotrowska@mail.umcs.pl; 4 Department of Virology and Immunology, Institute of Biological Sciences, Maria Curie-Skłodowska University, 20-031 Lublin, Poland; roman.paduch@mail.umcs.pl

**Keywords:** laccase, biocatalysis, bioactive compounds

## Abstract

Fungal laccase obtained from a *Cerrena unicolor* strain was used as an effective biocatalyst for the transformation of 8-anilino-1-naphthalenesulfonic acid into a green-coloured antibacterial compound, which can be considered as both an antimicrobial agent and a textile dye, simultaneously. The process of biosynthesis was performed in buffered solutions containing methanol as a co-solvent, allowing better solubilisation of substrate. The transformation process was optimised in terms of the buffer pH value, laccase activity, and concentrations of the substrate and co-solvent. The crude product obtained exhibited low cytotoxicity, antibacterial properties against *Staphylococcus aureus* and *Staphylococcus epidermidis*, and antioxidant properties. Moreover, the synthesised green-coloured compound proved non-allergenic and demonstrated a high efficiency of dyeing wool fibres.

## 1. Introduction

Laccase (LAC) is a versatile oxidiser of a wide range of compounds from simple phenols to more complex aromatic amines with a benzene, naphthalene, or anthraquinone framework. Thus, it is an important biocatalyst with enormous potential for industrial and biotechnological applications in the food and textile industries, organic synthesis, bio-sensors, biodegradation, and bioremediation [1,2,3,4,5]. Fungal laccase is a cheap and easy-to-handle catalyst, which can be obtained from liquid cultures of ligninolytic fungi. Laccase is involved in direct or mediator-driven oxidation of organic compounds with concomitant reduction of molecular oxygen to water, i.e., the purest co-substrate of the reaction. Despite the many years of research on its properties and application potential, the attention to laccase as a versatile catalyst for the synthesis of new chemical compounds with novel properties has not faded. The process of synthesis of new chemicals obtained by enzymatic oxidation has a low environmental impact and reduces the use of dangerous and often toxic oxidising compounds. Due to the low substrate specificity of laccase, novel and sometimes unexpected compounds are formed with unique physicochemical pro-perties, including textile dyes, bioactive compounds, antioxidants, antimicrobial agents, etc. [6,7,8]. The substrates for laccases are primarily represented by aromatic compounds with hydroxy, methoxy, and/or amino substituents, which facilitate laccase-mediated oxi-dation to radical forms that can undergo coupling reactions to dimers or polymers or internal rearrangements to often heterocyclic end products [9,10]. Laccase is involved in the formation of new bonds between easily oxidised natural substrates and different co-partner oxidation, leading to the formation of new hybrid molecules [9,10]. Mainly simple phenolic precursors can be found among the wide range of substrates for the laccase-mediated synthesis of bioactive compounds. Both products of their transformation and phenolic-functionalised compounds exhibit bioactive properties, e.g., antioxidative and antimicrobial activity [11,12]. However, aminophenolics represent a large group of substrates for laccase-mediated dye synthesis mainly due to the necessary participation of the amine group in the phenazine, phenoxazine, or indo dyes formation [13,14,15]. Sometimes, a large group of products obtained via laccase-mediated oxidation has been found to exhibit se-veral different activities or properties, for instance phenazine and phenoxazine dyes cha-racterised by both dyeing and bioactive properties, which enhance their application potential [15,16,17,18]. In addition to aminophenols, other more structurally complex chemicals such as azo dyes, i.e., common wastewater pollutants from the textile and dyeing industry, can also be considered dye precursors, as described by Enaud and co-workers, where laccase was an effective catalyst for the biotransformation of anthraquinonic dye Acid Blue 62 into a non-toxic red azo dye, namely LAR1 with novel dyeing properties [19]. 

The compound 8-anilinonaphthalenesulfonic acid (ANS) is a well-known light-green fluorescent dye used as a protein marker in molecular studies [20]. It is used as a probe to detect the hydrophobicity of protein regions, in studies of protein folding intermediates, and in research on the binding pockets of some carrier proteins [21,22,23]. ANS is an amphipathic molecule composed of a hydrophobic anilinonaphthalene group and a charged sulfonate group. Several schemes for the synthesis of ANS are known in the literature. Two of them developed in previous decades describe the reaction between 8-amino-1-naphthalenesulfonic acid and aniline catalysed by sulfuric acid or aluminium trichloride at temperatures above 155 °C for at least 10 h [24]. A more recent method describes the use of copper as a catalyst in microwave-assisted Ullmann coupling. The reaction proceeds in phosphate buffer as a solvent, making the process more environmentally friendly [25]. Thus far, only one paper has described the biotransformation of ANS by a culture of *Pseudomonas aeruginosa* into ketoadipic acid entering the TCA cycle [26]. Although ANS has been described in many papers as a possible precursor of dyes, to the best of our knowledge, no publications on its biotransformation by laccase have appeared until now. 

In the present work, the fluorescent dye ANS was used as a precursor for the synthesis of a novel potential green-coloured textile dye with new physicochemical and bioactive properties. The transformation of the substrate was optimised in terms of pH values, LAC activity, and methanol and substrate concentrations. In addition, the dyeing properties of the obtained non-purified dye were determined, as well as its toxicity and allergenicity and parameters with importance for the application safety. The dye was also evaluated for its antioxidant potential and its ability to inhibit the growth of *Staphylococcus aureus* ATCC^®^25923^TM^ and *Staphylococcus epidermidis* ATCC^®^14990^TM^ bacterial strains commonly occurring on the skin, indicating great applicability of the dye obtained.

## 2. Results and Discussion

### 2.1. Laccase-Mediated Transformation of 8-Anilinonaphthalenesulfonic Acid

Some of the many laccase-producing fungal species have been well recognised as the main source of laccase for applied biocatalysis [27]. There are numerous examples of fungal strains with the ability to synthesise laccase, but the most commonly mentioned in the context of biocatalysis are white-rot fungi such as *Trametes* sp., *Pleurotus* sp., or *C. unicolor* [10,28]. The cheap and easy-to-handle purified laccase obtained from the Basidiomycota fungus *C. unicolor* was applied for the homomolecular transformation of the 8-anilinonaphthalenesulfonic acid (ANS) into a novel water-soluble green-coloured dye. 

The ANS is a well-known fluorescent dye consisting of aniline attached to the naphthalene core with the sulfonic group at position 1. Despite the lack of a hydroxyl group in the structure of ANS, it was characterised by a low value of redox potential, which resulted in a low *K*_M_ value (Table 1), which is promising in terms of LAC-mediated oxidation and comparable to other fast oxidising substrates [29]. The nitrogen atom embedded in the imine group between the benzene and naphthalene is the only ring-activating substituent and may be crucial in the LAC-mediated transformation of ANS. In addition, the high oxygen consumption observed during the oxidation indicates that the substrate was rapidly and directly transformed by laccase without the involvement of any mediators. 

**Figure 1:**
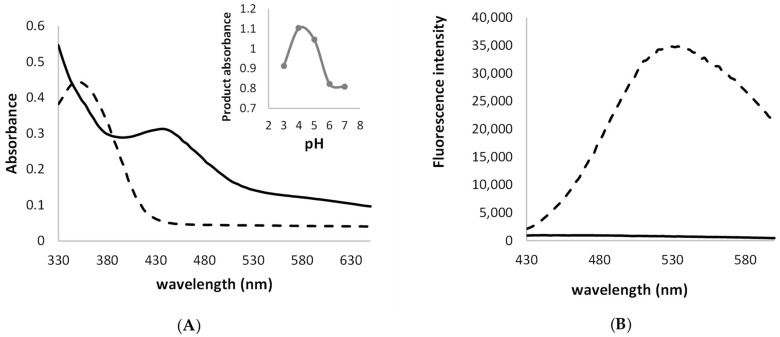
Absorption spectra of the ANS (dotted line) and dye (solid line) obtained at pH 4 after the LAC-mediated ANS oxidation with the optimum pH of oxidation reaction; (**A**) fluorescence spectra (**B**) of the ANS substrate (dotted line) in contrast to the lack of fluorescence of the crude dye obtained after the LAC-mediated oxidation of ANS (solid line) (excitation wavelength of 375 nm).

The effect of the pH value was slightly visible and exhibited a bell-shaped profile as in the case of phenolics as well as amine-substituting substrates with the maximum at pH 4 (Figure 1A) [15,30]. The laccase-mediated transformation of ANS resulted in the formation of a green water-soluble dye, which was characterised by maximum absorbance at a range from 420 to 440 nm (Figure 1A) and the loss of fluorescence in contrast to the substrate used (Figure 1B). 

**Figure 2:**
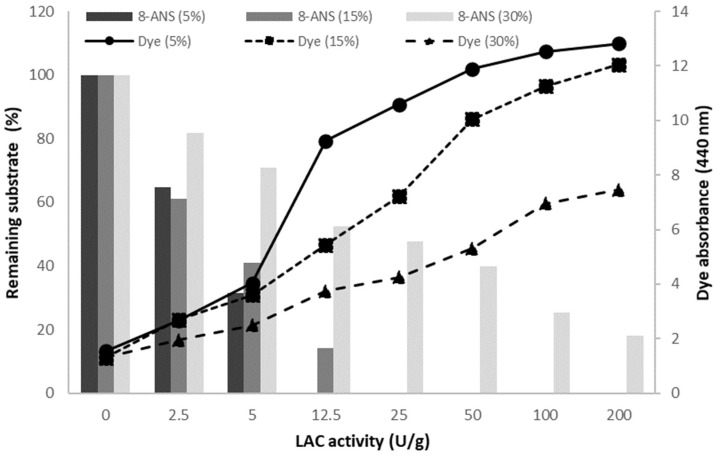
Effect of the methanol amount (in brackets: 5%, 15%, and 30% *v*/*v* total methanol concentration) and LAC activity (2.5–200 U/g: U per gram of ANS) on the yield of ANS oxidation (columns) and absorbance of the dye (lines) obtained after 24 h transformation of ANS (1 g/L) by LAC.

During the optimisation of the dye synthesis, methanol was used as a co-solvent, which allowed for the use of larger amounts of the substrates and thus improved the economic efficiency of the process. The dye obtained by homomolecular transformation of ANS with LAC was a mixture of at least three green-coloured compounds, and as such, was analysed for its synthesis and properties. The effect of the LAC activity and the me-thanol concentration on the amount of the dye obtained (expressed as absorbance at 440 nm) was evaluated for the substrate concentration of 1 g/L (Figure 2). In the case of transformation occurring in the presence of 5% methanol, the application of LAC with the activity of 12.5 U per 1 g of the substrate resulted in complete consumption of the substrate and 72% of maximum dye absorbance after 24 h of the reaction.

**Figure 3:**
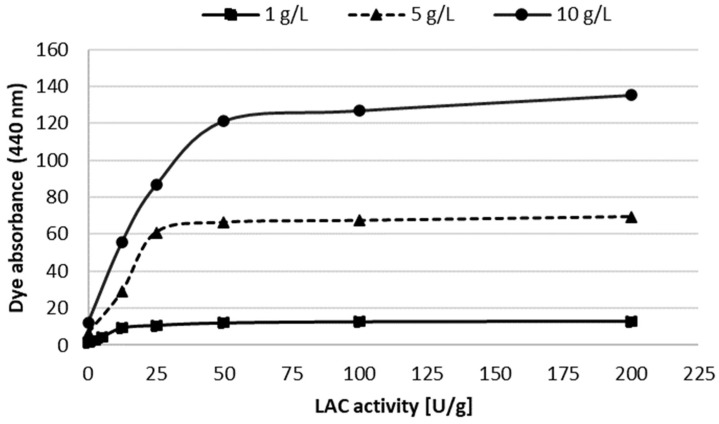
Effect of LAC activity (2.5–200 U/g: U per gram of ANS) on the absorbance of the dye obtained after the 24 h LAC-mediated oxidation of ANS different concentrations (1, 5 and 10 g/L) in a buffered transformation mixture containing 5% *v*/*v* methanol as a co-solvent.

The use of higher concentrations of methanol slowed down the rate of biotransformation of the substrate, which was probably caused by lower stability of LAC in organic solvents and which was reflected in the reduced amount of the dye obtained during the 24 h biotransformation. Higher amounts of ANS were possible to apply in the methanol-containing buffered reaction mixture (5%). When higher concentrations of the substrate were oxidised, the LAC activity determined the amount of the dye obtained (Figure 3). In the case of the 5 and 10 g/L concentrations of ANS, the application of 50 U/g LAC activity resulted in 95% and 90% of maximum dye absorbance, respectively. Considering the reduction of dye synthesis costs, such activity is economically justified. At all the tested ANS concentrations, no substrate remained after 24 h of the reaction, which was detected using the HPLC method. During the prolonged up to 48 h exposure to LAC, increasing absorbance of the dye was observed, which was visible, especially at the substrate concentrations of 5 and 10 g/L.

This phenomenon is consistent with the characteristics of LAC-mediated reactions. In the first step, the substrate is activated by LAC to form a radical, which in the second step, can be involved in a non-enzymatic coupling reaction to dimeric, oligomeric, or po-lymeric final products long after the substrate is no longer observed in the reaction medium [31].

### 2.2. Properties of the Dye

Modern compound synthesis technologies should be distinguished by environmental care and low costs, which can be based on impurified and non-toxic end products; therefore, the crude dye mixture was tested for its bioactivity, safety, and utility. 

The evaluation of environmental toxicity and cytotoxicity is an important step in the process of assessing the safety of chemical compounds. Azo dyes, aromatic amines, and their degradation products require special attention. There are a number of papers on the toxicity of azo dyes, where authors have defined three different mechanisms causing their carcinogenicity [32]. The first two mechanisms involve the metabolic oxidation of aromatic groups of dyes or free aromatic amines resulting from the reduction of the azo bond by intestinal anaerobic bacteria. Amine groups are metabolised to highly reactive electrophilic intermediates capable of covalent binding to DNA. The third mechanism involves oxidative activation of azo dyes to highly reactive electrophilic diazonium salts [32]. We found a strong impact of both tested compounds on the reduction of luminescence by the marine bacterium *Vibrio fisheri*, which is commonly used as an indicator of environmental toxicity (Table 2). 

This observation indirectly explains the ability of the tested compounds to be toxic for *Staphylococcus* strains. However, low cytotoxicity of both ANS and the dye tested with different methods was observed (Table 3). 

Regardless of the toxicity assay method and the cells used, the dye showed less to-xicity than the substrate with the values of IC_50_ not lower than 0.321 and 0.247 mg/mL, respectively. This may also be related to the presence of other non-toxic impurities such as tartaric acid and NaOH in the crude non-purified dye, which did not exceed 20% of total weight. In addition, the fibroblasts were shown to be more sensitive to both tested compounds than the colon epithelial cells. 

Some natural substrates for laccase-mediated oxidation are bioactive compounds with antimicrobial, antioxidative, or therapeutic properties that can be used as dyes, cosmetic additives, and substances for other biomedical applications [33]. The process of synthesis of valuable bioactive compounds can also be mediated by laccase as a catalyst. Se-veral compounds with antimicrobial activity were obtained by Mikolasch and co-workers [6,34,35]. An interesting group of compounds synthesised using laccase are dyes exhibiting various biological activities, which makes them valuable to industry [17]. Both ANS and the crude dye exhibited antibacterial potential against strains of the genus *Staphylococcus* that are commonly found on human skin and can cause skin diseases (Table 4). 

The ANS substrate was found to be more toxic to *S. aureus* cells than the product of its enzymatic oxidation. The MBC value increased more than twice after the transformation with LAC (from 0.4 mg/mL to 1 mg/mL) for this bacterial strain (Table 4). However, a different behaviour was observed in the case of *S. epidermidis* cells. This bacterium was found to be more sensitive to ANS (MIC value—0.025 mg/mL) than the *S. aureus* strain. Moreover, the laccase transformation product turned out to be much more toxic to *S. epidermidis* and caused a lethal effect at a concentration 0.1 mg/mL. 

As in the case of compounds with antimicrobial activity, laccase is a recognised catalyst of the synthesis of antioxidants as well. Phenolic compounds predominate among antioxidant structures, but other compounds deficient in hydroxyl groups, may also have antioxidant activity, such as some carotenoids, bilirubin, or melatonin [36]. An example is the use of laccase from *Trametes pubescens* as a catalyst for the transformation of hydroxytyrosol to dimers, oligomers, and polymers with enhanced antioxidant activities [37]. The antioxidative properties of compounds depend on the ability to interact with free radicals possessing an unpaired electron. There are several mechanisms of antioxidant action, including hydrogen atom transfer (HAT) and single electron transfer (SET), leading to damage or transformation of the radical [36]. Reduction of free radical species such as ABTS and DPPH by potential antioxidants is a commonly used method for evaluation of antioxidant activity [36]. The antioxidative properties of ANS and the crude dye were confirmed using three different methods (Table 5). 

Regardless of the method used, ANS exhibited higher antioxidative potential than the product of its transformation with IC_50_ values in the range from 0.027 to 153 µg/mL, depending on the method used. The antioxidant potential of the dye was lower at IC_50_ values, representing approximately five-fold higher concentrations of the compound compared to the substrate (Table 5).

The dye was tested to assess its dyeing potential and safety of use as a textile product. Wool dyed with the 1% concentration of the dye demonstrated high resistance to artificial light, alkaline and acidic sweat, and concomitant low resistance to washing at 40 °C (Table 6).

**Figure 4:**
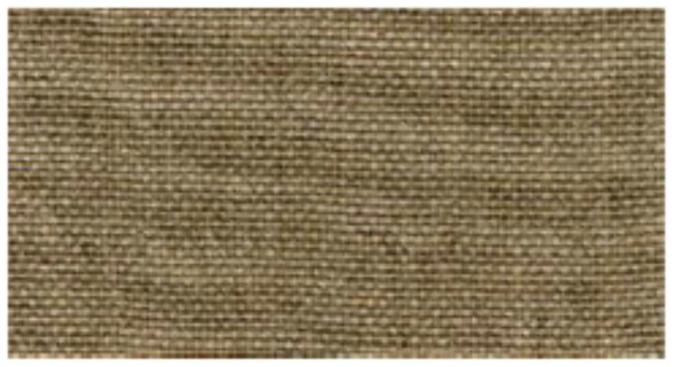
Sample of wool fabric dyed with 1% of the dye obtained through the LAC-mediated oxidation of ANS substrate.

Based on the results, it can be concluded that this compound exhibited promising potential for dyeing wool fibres, giving them a green-olive colour (Figure 4). 

The presence of a sulfonic group in the substrate structure, which is not involved in laccase reactions, indirectly indicates its presence in the reaction product, as confirmed by its high water solubility. A consequence is the observed high dyeing potential against wool through the possible interaction of the dye with the amino groups of wool, which was observed in the case of other dyes containing a sulfonic substituent [15]. As in the case of other chemicals, dyes should be characterised by low toxicity against human skin, evaluated using an allergenicity patch test. Therefore, the 1% solution of the crude dye and the wool fibre stained with the 1% dye showed no irritating and allergenic effects during dermatological tests on 50 volunteers, including those with increased skin sensitivity and regardless of their age and sex (Table 7). The results indicated the high commercial potential of the dye synthesised during the homomolecular transformation of 8-anilinonaphthalenesulfonic acid using fungal laccase as a natural biocatalyst into a non-irritating, antimicrobial green dye suitable for staining of wool.

## 3. Materials and Methods

### 3.1. Chemicals

Chemicals, i.e., tartaric acid and 8-anilinonaphthalenesulfonic acid ammonium salt (ANS), were purchased from Aldrich (Shanghai, China), and 2,2′-azino-bis(3-ethylbenzthiazoline-6-sulfonic acid (ABTS) was supplied by Sigma (St. Louis, MO, USA). All chemicals were of analytical grade and were used without further purification.

### 3.2. Preparations of Purified Laccase

The white rot fungus *C. unicolor* was the source of extracellular laccase (LAC). The strain (collection number FCL139) was obtained from the Fungal Collection of the Department of Biochemistry and Biotechnology of Maria Curie-Skłodowska University, Lublin (Poland). LAC was obtained and purified using a procedure described previously by Luterek and co-workers [38]. LAC activity was determined using ABTS as a substrate. Then, 50 µL of the LAC sample was added to the reaction mixture containing 200 µL of ABTS (2.5 mM final concentration) suspended in 750 µL of 0.1 M sodium-tartrate buffer pH 3. The oxidation of ABTS was monitored spectrophotometrically for one minute at 414 nm (λ_414_ = 36 048 M^−1^ cm^−1^) using Cary 50 Bio spectrophotometer (Varian, Palo Alto, CA, USA). The LAC activity was expressed in U/mL, where one unit (U) of the enzyme oxidised 1 µmol of ABTS per 1 min at 25 °C.

### 3.3. Substrate Characterisation

**Oxygen uptake** was detected during ANS transformation by LAC with a biological oxygen monitor (YSI model 5300). The standard vessel contained 3 mL of the transformation mixture containing 1 mM concentration of ANS dissolved in 0.1 M sodium-tartrate buffer pH 4.5. Each measurement was carried out for 3 min of the transformation and the oxygen uptake was calculated in nmol O_2_/mL/min according to Bernhardt [39]. 

**Cyclic voltammetry** measurement of the ANS substrate was carried out with a µAUTOLAB type III potentiostat/galvanostat (Metrohm Autolab B.V., Utrecht, The Netherlands) using a three-electrode cell containing a saturated Ag/AgCl/KCl_sat_ reference electrode, a platinum wire counter electrode, and a 2 mm diameter GC working electrode (all purchased from MTM-Anko, Kraków, Poland). All measurements were performed for the 1 mM substrate dissolved in 0.1 M sodium tartrate buffer at pH 4.5 in triplicate at room temperature 22 ± 3 °C. The potential was scanned with the scan speed of 50 mV/s from −400 to 1500 mV vs. Ag/AgCl/KCl_sat_ after holding the electrochemical system at the initial potential for 10 s. The measured potentials recorded vs. the Ag/AgCl/KCl_sat_ electrode were corrected by +0.199 V to the normal hydrogen electrode (NHE). 

**The kinetic constants *K*_M_** of the ANS were monitored at a wavelength of 440 nm in 0.1 M sodium-tartrate buffer pH 4 using a Cary 50 Bio spectrophotometer (Varian, Palo Alto, CA, USA) and were calculated using the Lineweaver–Burk equation. The optimum pH for LAC-mediated oxidation of ANS was determined in 100 mM sodium buffer with pH ranging from 3 to 7. The reaction mixture was scanned to determine the λ max cha-racterizing the main reaction product od dye using a Cary 50 Bio spectrophotometer (Varian, Palo Alto, CA, USA).

### 3.4. Laccase-Mediated Transformation of Substrate ANS—Optimisation Process

The laccase-mediated oxidation of ANS occurred in a 10 mL transformation mixture containing 100 mM tartrate buffer pH 4 and different amounts of methanol (5%, 15%, and 30% *v*/*v* final concentration). Different amounts of ANS (1, 5, and 10 g/L final concentrations) were oxidised by LAC with different final activities ranging from 12.5 to 200 U per gram of the ANS. The transformation mixtures were analysed using the HPLC method after 24 h to assess the optimal LAC activity for efficient substrate oxidation. Simultaneously, the absorbance of the product was monitored at 440 nm using a multiplate reader for maximum 96 h (Spark, Tecan, Grödig, Austria).

### 3.5. Large-Scale Synthesis of the Dye

In parallel with the optimisation studies, the synthesis of the N16 dye was performed in 1 L conical flasks containing 0.5 L reaction mixture. The transformation mixture contained the ANS substrate in the final concentration of 2 mM dissolved in ultrapure water with addition of 1 M NaOH. The pH value of the transformation mixture was adjusted to 4 using 100 mM of tartaric acid. In the last step, LAC was added to the mixture with the final activity of 1 U/mL. The transformation carried out in a rotary shaker (100 RPM) at 28 °C lasted 5 days. The absorbance of the synthesised N16 dye was measured spectrophotometrically in the range of 410–440 nm every day, and the transformation process was ended at the moment of plateau. The mixture of the dye was lyophilised and stored at 4 °C until use.

### 3.6. Characterisation of the Dye

**UV–visible spectra** of the substrate (ANS) and reaction mixtures containing dye were obtained on a Cary 50 Bio spectrophotometer (Varian, Pao Alto, CA, USA) or a multiplate reader (Spark, Tecan, Austria). The fluorescence intensity of ANS and the dye was measured in a 96-well plate using a multiplate reader. The sample was excited at 375 nm and the emitted light was measured in the range from 400 to 600 nm. 

**High performance liquid chromatographic** analyses were performed using an Agilent 1260 Infinity (Agilent^®^, Santa Clara, CA, USA) chromatograph coupled with a diode array detector. The biotransformation process of the ANS substrate was monitored by reverse-phase HPLC using a KNAUER column (Eurospher II, C18A, 3 × 25 mm, 3 µm). Methanol (eluent A) and 50 mM formate buffer adjusted to pH 4.1 using 1 M NaOH (eluent B) were used as eluents. The elution included an isocratic step with 50% *v*/*v* of eluent A for 1 min after injection of the sample; afterwards, a gradient step of elution (10 min) was applied in the range of 50–90% of eluent A. The separation was ended within 2 min of isocratic elution with 90% of eluent A. The total run time of each analysis was 13 min. After each analysis, a 5 min post run was conducted with 50% of eluent A to restore the start conditions of the analysis. The eluent flow rate was kept at 0.5 mL/min. throughout the separation process and the separation column was maintained at 40 °C. Each 2 µL sample was injected using an autosampler. The elution of compounds was monitored at a wavelength of 280 nm and 440 nm. Agilent OpenLAB CDS ChemStation LC and CE Drivers (A.02.10 (026) version) software was used for data processing and reporting. Identification of the substrate peak was achieved by comparing retention times with the standard.

### 3.7. Properties of Dye

#### 3.7.1. Toxicity

##### Environmental Toxicity

The environmental toxicity of the ANS substrate and the dye was assessed using the 81.9% Basic Test Microtox Protocol (https://www.modernwater.com/assets/Technical%20Support/Toxicity/Manuals/ACUTE%20User%27s%20Manual.pdf (accessed on 14 May 2020)) and using apparatus Microtox Model 500. Toxicity was expressed as half maximal effective concentration (EC_50_) according to the 81.9% Basic Test Microtox protocol. All reagents and *Vibrio fisheri* bacterium were purchased from Tigret (Warszawa, Polska), the Polish representative of Modern Water Company (New Castle, DE, USA).

##### Cytotoxicity

**Cell cultures:** The research was conducted on human normal colon epithelial cell line CCD-841 CoTr (ACCT, No. CRL-1807) and human normal colon fibroblast cell line CCD-18Co (ATCC, No. CRL-1459) obtained from the American Type Culture Collection (ATCC, Manassas, VA, USA). The cells were grown in a mixture of DMEM and RPMI 1640 media (1:1) containing 10% (*v*/*v*) of foetal bovine serum (FBS) and antibiotics (100 U/mL penicillin and 100 μg/mL streptomycin) and kept in standard conditions (at 37 °C in humidified atmosphere of 5% CO_2_). For the MTT and LDH tests, cells at the density of 1 × 10^5^ cells /mL were seeded on 96-well plates. After 24 h incubation, the medium was replaced with a fresh one with 2% of FBS supplemented with ANS or not (control).

**MTT assay:** This method is used to determine cell viability and proliferation. It is based on the measurement of the oxidoreductive activity of mitochondria with the yellow dye 3-(4,5-dimethylthiazo-2-yl)-2,5-diphenyl-tetrazolium bromide (MTT) which is metabolised in living cells to purple formazan crystals. After 24 h incubation with the examined compounds, 25 µL of MTT (5 mg/mL) was introduced onto each well and incubated for 3 h. Then, to stop the reaction and dissolve the formazan crystals, 100 µL of a 10 % SDS solution in 0.01 N HCL was added. The following day, the absorbance was determined spectrophotometrically at 570 nm using a microplate reader (Spark, Tecan, Austria). 

**Lactate dehydrogenase(LDH) assay:** This assay measures lactate dehydrogenase (LDH) released to the medium by cells with a damaged plasma membrane. An in vitro toxicology assay kit, Lactate Dehydrogenase (Sigma, USA and BioVision, Milpitas, CA, USA), was used to estimate LDH release according to the manufacturer’s instructions. After incubation, 50 µL of the culture supernatants from each well was transferred to a new 96-well multiplate. Next, 100 µL of the reaction solutions was introduced to all the wells and incubated for 30 min in darkness. To stop the enzyme reaction, 15 µL of 1 N HCl was added to each well. Absorbance was recorded on a microplate reader (Spark, Tecan, Austria) at 490 nm with a reference wavelength at 690 nm. 

Results obtained from three independent experiments were reported as cell viability (%) calculated according to the formula:

cell viability (%) = (100 − Abs_sample_ × Abs_control_^−1^) × 100%

All analyses were performed in triplicate and presented as mean ± SD. The IC_50_ value was calculated by nonlinear regression using a curve fitting program in GraphPad Prism 5.0. 

#### 3.7.2. Antimicrobial Properties

The Minimal Inhibitory Concentration (MIC) and the Minimal Bactericidal Concentration (MBC) of the ANS substrate and its transformation product were determined according to the CLSI Protocol [40] using *S. aureus* ATCC^®^ 25923^TM^ and *S. epidermidis* ATCC^®^ 14990^TM^ strains in a concentration range from 0.025 to 1 mg/mL. The stock solutions (10 mg/mL) of the tested substances were prepared in distilled water and afterwards diluted to the appropriate concentration in Mueller–Hinton Bullion. In each well of a 96-well plate, 300 µL of the mixture was inoculated with 10 µL of the bacterial culture (final density~10^2^ cfu/mL). After 24 h of incubation at 37 °C, the growth of the bacteria was controlled, and the MIC value was determined on the basis of the absence of bacterial growth by observation with the unaided eye. Then, 10 µL of the suspension from each well was transferred to a fresh sterile medium and incubated for another 24 h at 37 °C. The lack of turbidity indicated bactericidal activity of the investigated substances and the MBC value was thus determined.

#### 3.7.3. Antioxidant Properties

**Chemiluminescence Assay:** The assay of the antioxidant properties of the tested dyes was based on the measurement of luminol chemiluminescence induced by hydroxyl radicals in the Fe^2+^-EDTA-H_2_O_2_-luminol system. Samples of the dyes (100 µL) at concentrations ranging from 0.01 to 1 mg/mL were added to the mixture of 50 mM phosphate buffer pH 7.4 (600 µL) and 2 mM luminol in 95% ethanol (100 µL). Afterwards, the samples were placed in a Berthold–Lumat LB 9506 luminometer, and the solutions of reagents were added automatically as follows: 1.5 mM Fe^2+^-EDTA (100 µL) and 4.4 mM H_2_O_2_ (100 µL). The chemiluminescence was measured with (*I*_1_) or without (*I*_0_) the addition of the tested dye. The inhibitory rate (*I_R_*) was calculated according to the following equation:

$$I_{R}\left(\%\right)=\left(1-\frac{I_{1}}{I_{o}}\right)\times 100$$

**DPPH assay:** This method is based on the ability of the tested dyes to reduce the dark purple 2,2-diphenyl-1-picrylhydrazyl radical (DPPH) to the yellow diphenyl-picrylhydrazine. Then, 100 µL of the tested dye was added to a 96-well plate containing 100 µL of a freshly prepared DPPH solution in 95% ethanol. The concentration of DPPH was 0.2 mg/mL, and the tested dye concentration was in the range from 0.01 to 1 mg/mL. The mixture was incubated at room temperature for 20 min. The change in absorbance caused by the tested dyes (*X_P_*) and ultrapure water as a negative control (*X_C_*) was monitored at 515 nm using a multiplate reader (Spark, Tecan, Austria). Simultaneously, the absorbance of the dye at a wavelength of 515 nm was investigated as a control of the dye–colour correction (*X_D_*). The radical reduction rate (*D_R_*) was calculated according to the following equation:

$$D_{R}\left(\%\right)=\left(\frac{X_{C}-\left(X_{P}-X_{D}\right)}{X_{C}}\right)\times 100\%$$

**ABTS assay:** In this method, the ability of the tested dye to reduce the dark green ABTS radical cation (ABTS^●+^) was evaluated. First, 5 mM phosphate buffer pH 7.4 (PBS) and 7.4 mM ABTS solution were prepared. During the ABTS solution preparation, ABTS was dissolved and ABTS^●+^ was generated by reacting with K_2_S_2_O_8_ (2.4 mM final concentration) in a dark bottle at room temperature for 16–18 h. Next, the ABTS^●+^ solution was diluted with PBS to an absorbance of 0.7 at 734 nm measured using a Tecan plate reader (Spark, Tecan, Austria). ABTS^●+^ (990 µL) was added to the tested dye sample (10 µL) in a 96-well plate and incubated for 10 min at room temperature. The tested dye concentration was in the range from 0.01 to 1 mg/mL. Simultaneously, the negative control (*X_C_*) and the control of dye colour correction (*X_D_*) were incubated. The change in the absorbance of ABTS^●+^ with the dye sample (*X_P_*) and controls were measured in the plate reader at 734 nm and the ABTS^●+^ reduction rate (*A_R_*) was calculated according to the following equation:

$$A_{R}=\left(\frac{X_{C}-\left(X_{P}-X_{D}\right)}{X_{C}}\right)\times 100\%$$

All analyses allowing determination of antioxidative properties were performed in triplicate in room temperature 22 ± 3 °C. The antioxidative potential of the ANS and the dye was expressed as IC_50_, which represents the final concentration of the tested compound (µg/mL) that reduces 50% of the radical amounts. Trolox was used as positive controls for each technique.

#### 3.7.4. Dyeing Properties

Wool fabric dyed using 1% solution of crude dye was tested under its colour stability during fastness test prepared and analysed by the TKANLAB Laboratory (Łódź, Poland). The fibre characteristics are as follows: yarn linear mass—warp R63 ± 4/2 weft R74 ± 4/2; number of threads per 10 cm—warp 175 ± 10, weft 135 ± 8; mass per unit area—215 ± 10 g/m^2^; fat content—0.8 ± 0.3%; linen weave.

#### 3.7.5. Allergenicity—Patch Test

The study was conducted in accordance with Regulation No 1223/2009 of the European Parliament and of the Council (EC) of 30 November 2009 on cosmetics and with the recommendation of Cosmetics Europe—The Personal Care Association Guidelines [41,42]. The research was carried out in compliance with internal procedures of Dr. Koziej Institute Ltd. Patch tests according to Jadassohn–Bloch with Rudzki modifications were conducted under careful supervision of a medical specialist—dermatologist. The assessment of the allergenic and irritant features was carried out in a group of 50 healthy volunteers with positive allergic screening. The subjects were obliged to follow all the guidelines included in the procedures of particular carefulness during the study. The study was conducted to determine local skin tolerance to the crude non-purified dye in a group of 50 skin-healthy volunteers aged 19–70 (34 women and 16 men) with increased sensitivity and positive allergenic screening of the skin and to establish possible irritant and/or sensitising properties of the product. The selection of probands—volunteers was conducted by a dermatologist according to the Declaration of Helsinki of 1964 (with subsequent amendments), Polish laws, and Cosmetics Europe directives with application of inclusion and exclusion criteria. All the volunteers were familiar with the procedure of the study and signed conscious consent to take part in the study. The application of the patch tests was preceded by a health survey including information on the present and former illnesses, a survey on coexisting skin problems (allergy issues included), dermatology examination, and assessment of the type of skin and the presence of any pathological changes in the skin. The skin where the patch tests were applied was healthy and free of any lesions. The subjects were informed not to expose their skin or take any anti-histamine or other pharmaceutical drugs (both systemic and local) that could come into interference with the applied product and have an influence on the results of the study. Two kinds of samples were prepared: the first was the dye diluted to 1% and applied to the patch, and the second was material stained with the tested dye cut into the size of the chamber and placed on the patch. The study was conducted using Finn Chambers on Scanpor 100 × 10 8 mm. The whole patch was then applied to the volunteer’s skin (shoulders, arm, or back). The patches were removed after 15 min. The patch tests were assessed after 48 and 72 h after patch removal.

## 4. Conclusions

Sustainable synthesis processes involving a versatile natural catalyst such as fungal laccase are the future for new possible applications. The potential of laccase is associated with the non-obvious substrates used for the transformation, which increase the number of possible new reaction products with unique properties. Mild reaction media, oxygen as a co-substrate, no-waste technology, and a solvent-tolerant catalyst are the main advantages of using laccase as a biocatalyst in the eco-friendly synthesis of chemicals, including bioactive compounds. Integrating different activities and properties in the product of laccase action is highly promising for its potential applications.

## Figures and Tables

**Table 1:** Characteristics of the main catalytic parameters of 8-anilino-1-naphthalenesulfonic acid (ANS) as the potential substrate in terms of LAC-mediated oxidation.

Chemical Structure	Oxygen Demand (nM/min/mL)	Eo vs. NHE (V)	Optimal pH	*K*_M_ (mM)	λ max^1^ (nm)	λ max^2^ (nm)
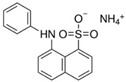	1583	0.768	4	0.435	340–350	420–440

Eo, oxidation potential; *K*_M_, Michaelis–Menten constant; λ max^1^, absorption maximum wavelength of ANS; λ max^2^, absorption maximum wavelength of product.

**Table 2:** Environmental toxicity of the ANS substrate and the dye after 5 min of exposure on *V. fisheri* bacteria, expressed as half maximal effective concentration (EC_50_) according to the 81.9% Basic Test Microtox protocol.

Tested Compounds	EC_50_ (mg/mL)
ANS	0.019
Dye	0.067

**Table 3:** Cytotoxicity of the ANS substrate and the dye expressed as IC_50_ (mg/mL) after 24 h using colon epithelial cells and fibroblasts tested with LDH and MTT assays.

Tested Compounds	Colon Epithelial Cells		Fibroblasts	
	LDH	MTT	LDH	MTT
ANS	0.382	0.322	0.247	0.295
Dye	0.436	0.473	0.321	0.363

**Table 4:** Antibacterial potential of the tested substrate (ANS) and dye expressed in mg/mL as the minimal inhibitory concentration (MIC) and the minimal bactericidal concentration (MBC).

Tested Compounds	*S. aureus* (0.51 × 10^2^ cfu/mL)		*S. epidermidis* (0.05 × 10^2^ cfu/mL)		Inhibition Zone Caused by Dye (*S. aureus*)
	MIC	MBC	MIC	MBC	
ANS	0.2	0.4	0.025	0.4	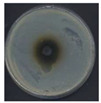
Dye	0.4	1.0	0.025	0.1	

**Table 5:** Antioxidative potential of the ANS substrate and the dye obtained using different methods expressed as the effective concentration (µg/mL) of the tested compounds reducing 50% of radicals (IC_50_).

Tested Compounds	Method		
	ABTS	DPPH	Luminol
ANS	0.027	153	6
Dye	0.115	660	42

**Table 6:** Colour fastness of wool fibres dyed with the use of the dye obtained through the LAC-mediated ANS oxidation.

Tested Parameters of Colour Fastness (ISO Standard)		Scale *
**Artificial light** ^(1)^ (PN-EN ISO 105-B02:2014-11)	(a)	6–7
**Distilled water** ^(2)^ (PN-EN ISO 105-E01:2013-06)	(a)	4
	(b)	4–5
	(c)	4
**Washing 40 °C** ^(2)^ (PN-EN ISO 105-C06:2010)	(a)	2
	(b)	4–5
	(c)	4–5
**Alkaline sweat** ^(2)^ (PN-EN ISO 105-E04:2013-06)	(a)	4–5
	(b)	3–4
	(c)	3
**Acidic sweat** ^(2)^ (PN-EN ISO 105-E01:2013-06)	(a)	4–5
	(b)	4–5
	(c)	4
**Dry rubbing** ^(2)^ (PN-EN ISO 105-X12:2005)	(b)	3
**Wet rubbing** ^(2)^ (PN-EN ISO 105-X12:2005)	(b)	3–4

* Colour fastness according to a blue ^(1)^ or grey ^(2)^ scale in which index “8” or “5” respectively means the highest resistance and “1” means the lowest resistance according to PN-EN20105-A02:1996 and PN-EN 20105-A03:1996 standards; (a) change in the colour of the tested sample; (b) soiled whiteness of the accompanying fabric—cotton; (c) soiled whiteness of the accompanying fabric—wool.

**Table 7:** Effect of the dye obtained through the LAC-mediated ANS transformation on human skin.

Probant No.		1	2	3	4	5	6	7	8	9	10	11	12	13	14	15	16	17	18	19	20	21	22	23	24	25	26	27	28	29	30	31	32	33	34	35	36	37	38	39	40	41	42	43	44	45	46	47	48	49	50
**Sex ^(1)^**		M	F	F	F	F	F	F	F	F	M	M	F	F	F	F	F	M	F	F	F	M	M	M	F	M	M	F	M	F	M	F	M	M	M	M	F	F	F	F	F	F	F	F	F	F	M	F	F	F	F
**Age**		33	28	50	54	42	39	26	70	49	51	70	33	19	53	22	22	30	23	50	70	40	69	23	70	36	65	70	40	37	40	20	67	38	69	66	33	65	35	40	31	40	41	24	24	35	27	54	31	31	29
**Skin type ^(2)^**		S	S	S	SA	S	S	S	S	S	SA	S	S	S	S	S	S	SA	S	S	S	S	S	S	S	S	S	S	S	S	S	S	S	S	S	S	S	S	S	S	S	S	S	S	S	S	S	S	SA	SA	S
**Skin response (3)**	**48 h**		-	-	-	-	-	-	-	-	-	-	-	-	-	-	-	-	-	-	-	-	-	-	-	-	-	-	-	-	-	-	-	-	-	-	-	-	-	-	-	-	-	-	-	-	-	-	-	-	-
	**72 h**		-	-	-	-	-	-	-	-	-	-	-	-	-	-	-	-	-	-	-	-	-	-	-	-	-	-	-	-	-	-	-	-	-	-	-	-	-	-	-	-	-	-	-	-	-	-	-	-	-

^(1)^ F, female, M, male; ^(2)^ S, sensitive, SA, sensitive and allergenic; ^(3)^ “-”, no irritating or allergenic effect.

## Sample Availability

Samples of the compounds are available from the authors.

## References

[1] Pezzella C., Guarino L., Piscitelli A. (2015). How to enjoy laccases. Cell Mol. Life Sci..

[2] Widsten P., Kandelbauer A. (2008). Laccase applications in the forest products industry: A review. Enzym. Microb. Technol..

[3] Mate D.M., Alcade M. (2017). Laccase: A multi-purpose biocatalyst at the forefront of biotechnology. Microb. Biotechnol..

[4] Pozdnyakova N., Jarosz-Wilkolazka A., Polak J., Wlizlo K., Dubrovskaya E., Turkovskaya O., Harris A. (2017). Unique properties of fungal laccases for biodegradative processes. Laccase: Applications, Investigations and Insights.

[5] Mayolo-Deloisa K., González-González M., Rito-Palomares M. (2020). Laccases in food industry: Bioprocessing, potential industrial and biotechnological applications. Front. Bioeng. Biotechnol..

[6] Mikolasch A., Manda K., Schlüter R., Lalk M., Witt S., Seefeldt S., Hammer E., Schauer F., Jülich W.D., Lindequist U. (2012). Comparative analyses of laccase-catalyzed amination reactions for production of novel β-lactam antibiotics. Biotechnol. Appl. Biochem..

[7] Pezzela C., Giacobbe S., Giacobelli V.G., Guarino L., Kylic S., Sener M., Sannia G., Piscitelli A. (2016). Green routes towards industrial textile dyeing: A laccase based approach. J. Mol. Catal. B Enzym..

[8] Kudanga T., Nemadziva B., Le Roes-Hill M. (2017). Laccase catalysis for the synthesis of bioactive compounds. Appl. Microbiol. Biotechnol..

[9] Bassanini I., Ferrandi E.E., Riva S., Monti D. (2021). Biocatalysis with laccases: An updated overview. Catalysts.

[10] Sousa A.C., Martins L.O., Robalo M.P. (2021). Laccases: Versatile biocatalysts for the synthesis of heterocyclic cores. Molecules.

[11] Adelakun O.E., Kudanga T., Parker A., Green I.R., le Roes-Hill M., Burton S.G. (2012). Laccase catalyzed dimerization of ferulic acid amplifies antioxidant activity. J. Mol. Catal. B Enzym..

[12] Rocasalbas G., Francesko A., Touriño S., Fernández-Francos X., Guebitz G.M., Tzanov T. (2013). Laccase-assisted formation of bioactive chitosan/gelatin hydrogel stabilized with plant polyphenols. Carbohydr. Polym..

[13] Sousa A.C., Conceição Oliveira M., Martins L.O., Robalo M.P. (2014). Towards the rational biosynthesis of substituted phenazines and phenoxazinones by laccases. Green Chem..

[14] Sousa A.C., Piedade M.F.M., Martins L.O., Robalo M.P. (2016). Eco-friendly synthesis of indo dyes mediated by a bacterial laccase. Green Chem..

[15] Polak J., Wlizło K., Pogni R., Petricci E., Grąz M., Szałapata K. (2020). Structure and bioactive properties of novel textile dyes synthesised by fungal laccase. Int. J. Mol. Sci..

[16] Forte S., Polak J., Valensin D., Taddei M., Basosi R., Vanhulle S., Jarosz-Wilkołazka A., Pogni R. (2010). Synthesis and structural characterization of a novel phenoxazinone dye by use of a fungal laccase. J. Mol. Catal. B Enzym..

[17] Polak J., Jarosz-Wilkołazka A., Szałapata K., Grąz M., Osińska-Jaroszuk M. (2016). Laccase-mediated synthesis of a phenoxazine compound with antioxidative and dyeing properties–the optimisation process. New Biotechnol..

[18] Sousa A.C., Oliveira M.C., Martins L.O., Robalo M.P. (2018). A sustainable synthesis of asymmetric phenazines and phenoxazinones mediated by CotA-laccase. Adv. Synth. Catal..

[19] Enaud E., Trovaslet M., Bruyneel F., Billottet L., Karaaslan R., Sener M.E., Coppens P., Casas A., Jaeger I.J., Hafner C. (2010). A novel azoanthraquinone dye made through innovative enzymatic process. Dyes Pigment..

[20] Robinson G.W., Robbins R.J., Fleming G.R., Morris J.M., Knight A.E.W., Morrison R.J.S. (1978). Picosecond studies of the fluorescence probe molecule 8-anilino-1-naphthalenesulfonic acid. J. Am. Chem. Soc..

[21] Gasymov O.K., Abduragimov A.R., Glasgow B.J. (2008). Ligand binding site of tear lipocalin: Contribution of a trigonal cluster of charged residues probed by 8-anilino-1-naphthalenesulfonic acid. Biochemistry.

[22] Singh K., Hussain I., Mishra V., Md Akhtar S. (2019). New insight on 8-anilino-1-naphthalene sulfonic acid interaction with TgFNR for hydrophobic exposure analysis. Int. J. Biol. Macromol..

[23] Ota C., Tanaka S.-I., Takano K. (2021). Revisiting the rate-limiting step of the ANS–protein binding at the protein surface and inside the hydrophobic cavity. Molecules.

[24] Wu F., Hu Z., Zhang Y., Cheng L. (2016). Preparation Method of N-Phenyl-8-Naphthylamine-1-Sulfonic Acid. CN Patent.

[25] Wang N., Faber E.B., Georg G.I. (2019). Synthesis and spectral properties of 8-anilinonaphthalene-1-sulfonic acid (ANS) derivatives prepared by microwave-assisted copper(0)-catalyzed Ullmann reaction. ACS Omega.

[26] Nachiyar C.V., Rajakumar G.S. (2006). Biodegradation of 8-anilino-1-naphthalenesulfonic acid by *Pseudomonas aeruginosa*. J. Ind. Microbiol. Biotechnol..

[27] Moreno A.D., Ibarra D., Eugenio M.E., Tomás-Pejóc E. (2020). Laccases as versatile enzymes: From industrial uses to novel applications. Chem. Technol. Biotechnol..

[28] Janusz G., Pawlik A., Świderska-Burek U., Polak J., Sulej J., Jarosz-Wilkołazka A., Paszczyński A. (2020). Laccase properties, physiological functions, and evolution. Int. J. Mol. Sci..

[29] Polak J., Jarosz-Wilkołazka A. (2012). Structure/redox potential relationship of simple organic compounds as potential precursors of dyes for laccase-mediated transformation. Biotechnol. Prog..

[30] Xu F. (1997). Effects of redox potential and hydroxide inhibition on the pH activity profile of fungal laccases. J. Biol. Chem..

[31] Wlizło K., Polak J., Jarosz-Wilkołazka A., Pogni R., Petricci E. (2020). Novel textile dye obtained through transformation of 2-amino-3-methoxybenzoic acid by free and immobilised laccase from a *Pleurotus ostreatus* strain. Enzym. Microb. Technol..

[32] Brown M.A., De Vito S.C. (1993). Predicting azo dye toxicity. Crit. Rev. Environ. Sci. Technol..

[33] Patel H., Gupte A. (2019). Laccase catalysis: A green approach in bioactive compound synthesis. Research Advancements in Pharmaceutical, Nutritional, and Industrial Enzymology.

[34] Mikolasch A., Wurster M., Lalk M., Witt S., Seefeldt S., Hammer E., Schauer F., Jülich W.D., Lindequist U. (2008). Novel beta-lactam antibiotics synthesized by amination of catechols using fungal laccase. Chem. Pharm. Bull..

[35] Mikolasch A., Hildebrandt O., Schlüter R., Hammer E., Witt S., Lindequist U. (2016). Targeted synthesis of novel β-lactam antibiotics by laccase-catalyzed reaction of aromatic substrates selected by pre-testing for their antimicrobial and cytotoxic activity. Appl. Microbiol. Biotechnol..

[36] Santos-Sánchez N.F., Salas-Coronado R., Villanueva-Cañongo C., Hernández-Carlos B. (2019). Antioxidant compounds and their antioxidant mechanism. Antioxidants.

[37] Burton S.G., Davids L.M. (2014). Hydroxytyrosol Compounds. U.S. Patent.

[38] Luterek J., Gianfreda L., Wojtaś-Wasilewska M., Rogalski J., Jaszek M., Malarczyk E., Dawidowicz A., Ginalska G., Leonowicz A., Finks-Boots M. (1997). Screening of the wood rotting fungi for laccase production: Induction by ferulic acid, partial purification, and immobilization of laccase from the high laccase-producing strain, *Cerrena unicolor*. Acta Microbiol. Pol..

[39] Bernhardt F.-H., Staudinger H., Ulrich V. (1970). The properties of p-anisate O-demethylase in cell-free extracts of *Pseudomonas* sp. Hoppe-Seyler’s Z. Physiol. Chem..

[40] (2012). CLSI Method for dilution antimicrobial susceptibility testing for bacteria that grow aerobically. Approved Standard.

[41] (1997). Product Test Guidelines for the Assessment of Human Skin Compatibility. https://www.cosmeticseurope.eu/files/6014/6407/8875/Product_Test_Guidelines_for_the_Assessment_of_Human_Skin_Compatibility_-_1997.pdf.

[42] (2008). Guidelines for the Evaluation of the Efficacy of Cosmetic Products. https://cosmeticseurope.eu/files/4214/6407/6830/Guidelines_for_the_Evaluation_of_the_Efficacy_of_Cosmetic_Products_-_2008.pdf.

